# Dynamic evolution of MADS-box genes in extant ferns via large-scale phylogenomic analysis

**DOI:** 10.3389/fpls.2024.1410554

**Published:** 2024-06-21

**Authors:** Rui Zhang, Jiao Zhang, Yue-Xia Xu, Jun-Mei Sun, Shao-Jun Dai, Hui Shen, Yue-Hong Yan

**Affiliations:** ^1^ Eastern China Conservation Centre for Wild Endangered Plant Resources, Shanghai Chenshan Botanical Garden, Shanghai, China; ^2^ College of Life Science, Shanghai Normal University, Shanghai, China; ^3^ School of Science, Qiongtai Normal University, Haikou, Hainan, China; ^4^ Shenzhen Key Laboratory for Orchid Conservation and Utilization, National Orchid Conservation Center of China and the Orchid Conservation and Research Center of Shenzhen, Shenzhen, China

**Keywords:** MIKC, ferns, most recent common ancestor, MADS-box, phylogenetic analysis

## Abstract

**Introduction:**

Several studies of MADS-box transcription factors in flowering plants have been conducted, and these studies have indicated that they have conserved functions in floral organ development; MIKC-type MADS-box genes has been proved to be expanded in ferns, however, few systematic studies of these transcription factors have been conducted in non-seed plants. Although ferns and seed plants are sister groups, they exhibit substantial morphological differences.

**Methods:**

Here, we clarified the evolution of MADS-box genes across 71 extant fern species using available transcriptome, genome, and gene expression data.

**Results:**

We obtained a total of 2,512 MADS-box sequences, ranging from 9 to 89 per species. The most recent common ancestor (MRCA) of ferns contained approximately three type I genes and at least 5–6 type II MADS-box genes. The domains, motifs, expression of type I and type II proteins, and the structure of the both type genes were conserved in ferns as to other land plants. Within type II genes, MIKC*-type proteins are involved in gametophyte development in ferns; MIKC^C^-type proteins have broader expression patterns in ferns than in seed plants, and these protein sequences are likely conserved in extant seed plants and ferns because of their diverse roles in diploid sporophyte development. More than 90% of MADS-box genes are type II genes, and MIKC^C^ genes, especially CRM1 and CRM6-like genes, have undergone a large expansion in leptosporangiate ferns; the diverse expression patterns of these genes might be related to the fuctional diversification and increased complexity of the plant body plan. Tandem duplication of CRM1 and CRM6-like genes has contributed to the expansion of MIKC^C^ genes.

**Conclusion or Discussion:**

This study provides new insights into the diversity, evolution, and functions of MADS-box genes in extant ferns.

## 1 Introduction

MADS-box genes are critically important transcription factors involved in diverse processes, including all major aspects of sporophyte and gametophyte development in land plants (Gramzow and Theissen, 2010; Qiu et al., 2023). They are widespread in eukaryotes including fungi, animals, and plants (Norman et al., 1988; Passmore et al., 1988; Sommer et al., 1990; Yanofsky et al., 1990; Gramzow and Theissen, 2010). The “M,” “A,” “D,” and “S” in MADS are derived from the first letters of *MCM1* from yeast (*Saccharomyces cerevisiae*), *AG* from *Arabidopsis*, *DEF* from snapdragon (*Antirrhinum majus*), and *SRF* from humans (*Homo sapiens*), respectively (Schwarz-Sommer et al., 1990). MADS-box genes have been classified into type I and type II two major groups in extant eukaryotes (Alvarez-Buylla et al., 2000; Gramzow and Theissen, 2010).

Type I proteins in plants can be further divided into the Mα, Mβ, and Mγ subgroups (Parenicová et al., 2003; Arora et al., 2007; Gramzow and Theissen, 2010). Few studies of the functions of type I proteins in plants have been conducted, and these studies have indicated that these proteins play key roles in the development of the female gametophyte, embryo sac, and seeds via analysis of mutants (Kohler et al., 2003; Colombo et al., 2008; Gramzow and Theissen, 2010). Type II MADS-domain proteins comprise two subgroups: MIKC^C^ and MIKC* (Kwantes et al., 2012; Theißen and Gramzow, 2016). MIKC* genes are mainly expressed in the gametophyte or in the pollen (male gametophyte) of flowering plants, suggesting their conserved role in the haploid phase during land plant evolution (Zobell et al., 2010; Kwantes et al., 2012; Liu et al., 2013). According to the classic ABCDE model of floral development, MIKC^C^-type genes and *AP2* are the main regulators of floral organ identity, and these genes are known to have specific conserved functions in floral morphogenesis in flowering plants (Theißen and Saedler, 2001; Zahn et al., 2006; Causier et al., 2010; Gramzow and Theissen, 2010). MIKC^C^ genes might have more ubiquitous functions through their effects on various aspects of diploid sporophyte development in land plants; these genes also regulate development and cell differentiation in green algae and early land plants (Theissen et al., 2000; Tanabe et al., 2005); flowering time determination (Adamczyk et al., 2007; Lee et al., 2007; Liu et al., 2008); the development of seeds, embryos, and fruit (Liljegren et al., 2000; Nesi et al., 2002); and leaf and root morphogenesis (Tapia-López et al., 2008; Zhang et al., 2017).

Land plants have a much higher number of MADS-box genes than green algae, animals, or fungi (Gramzow and Theissen, 2010; Theißen and Gramzow, 2016). The expansion of MADS-box family genes in land plants might be linked to complexity and diversification in the body structure of land plants (Kaufmann et al., 2005; Thangavel and Nayar, 2018; Qiu et al., 2023). Land plants exhibit dramatic morphological diversity; they evolved from unicellular green algae, seed-free plants have simple body plans; seed plants eventually became dominant, given their ability to withstand variable environments (Niklas et al., 1983; Crane et al., 1995; Cronk, 2001; Schuettpelz and Pryer, 2009; Cheng et al., 2019). Ferns (Monilophyta) are sister to seed plants; a total of 10,578 fern species have been described to date, which constitute the second largest group of vascular plants. They occupy a key phylogenetic position in the plant phylogeny and show significant morphological differences with their closest relatives (Theissen et al., 2000; Schuettpelz and Pryer, 2007; PPGI, 2016). Hence, systematic studies of MADS-box genes in non-seed vascular plants, such as ferns, and comparative analysis of these genes in various types of plants can enhance our understanding of their roles in morphological innovation and the evolutionary success of land plants. However, only a few studies of MADS-box genes in fern species have been conducted (Münster et al., 1997; Hasebe et al., 1998; Münster et al., 2002; Huang et al., 2014; Ruiz-Estévez et al., 2017; Thangavel and Nayar, 2018; Leebens-Mack et al., 2019; Zhang et al., 2019); thus, little is known about MADS-box genes in ferns and their potential functions. Hence, analyses with dense taxon sampling of MADS-box genes have become urgent to fill the gap in the evolutionary history of MADS-box.

The sequencing of fern genomes, especially homosporous fern genomes, has been hindered by their large size and high number of chromosomes. Only four homosporous ferns have been sequenced to date (Fang et al., 2022; Huang et al., 2022; Marchant et al., 2022; Zhong et al., 2022). Transcriptome sequencing (RNA-Seq) has become an efficient method for generating abundant sequence information at a low cost without a reference genome (Barker and Wolf, 2010). Sixty-nine fern species representing all major fern lineages were sequenced based on high-quality transcriptome sequencing data to construct a large-scale fern phylogeny (Shen et al., 2018). Here, we conducted a systematic analysis of MADS-box family genes using large-scale transcriptome data and few complete fern genomes available. Next, the locations of MADS-box genes were determined from the *Ceratopteris richardii* genome to elucidate gene family expansions. Spatial variation in the expression of MADS-box genes in *Lygodium japonicum* was examined to clarify the potential functions of MADS-box genes in ferns. Our findings enhance our understanding of the evolution of MADS-box genes during the origin and diversification of land plants.

## 2 Materials and methods

### 2.1 Data collection

Data were obtained from a total of 71 extant fern species from 38 families (including eusporangiate ferns, early leptosporangiates, and Polypodiales) and lycophytes (**Supplementary Table S1**). Transcriptome data from 69 fern species and three lycophytes were downloaded from the GigaScience repository, GigaDB and the Genome Sequence Archive in National Genomics Data Center, China National Center with accession number CRA00961 at https://ngdc.cncb.ac.cn/gsa/ (Shen et al., 2018; Xia et al., 2022). Transcriptome data for *L. japonicum*, including gene expression profiles, were obtained from the *L. japonicum* transcriptome database at http://bioinf.mind.meiji.ac.jp/kanikusa/ (Aya et al., 2015). The whole-genome assembly of *C. richardii* was downloaded from https://www.ncbi.nlm.nih.gov/datasets/genome/GCA_020310875.1/ (Marchant et al., 2022).

### 2.2 Identification of MADS-box family genes

MADS-box proteins for *Chara globularis*, *Selaginella moellendorffi*, *Physcomitrella patens*, and *Arabidopsis* were from the Phytozome database (http://www.phytozome.net), NCBI (http://blast.ncbi.nlm.nih.gov/) online resources, and the Plant Transcription Factor Database (http://plntfdb.bio.uni-potsdam.de/v3.0/) according to previous studies (Zhang et al., 2019). The SRF-TF domain (PF00319) seed file from the Pfam database (http://pfam.xfam.org/) was obtained, and the HMMER3.0 software package was used to determine new MADS-box genes across the 74 species (http://hmmer.janelia.org/) (Finn et al., 2016). Additionally, BLASTP searches were conducted to identify protein transcripts using Arabidopsis MADS proteins as query sequences with the cutoff value of 1e −5. The NCBI CDD was used to confirm all putative MADS-box genes obtained and remove incomplete MADS-box domains.

### 2.3 Gene structure, conserved motifs and domains analysis of MADS-box genes

The exon–intron structure of each MADS-box gene was predicted based on the annotated *C. richardii* genome. The conserved motifs of MADS-box genes were identified using the MEME v5.5.5 online tool (http://meme-suite.org/tools/meme), with the maximum number of motifs as 10. The conserved domains were predicted using the CDD. TBtools v2.034 was used to visualize the results (Chen et al., 2020).

### 2.4 Phylogenetic analysis and classification of MADS-box family genes

The identified MADS-box genes for each species were classified into type I and type II groups based on the classification of MADS-box genes in *A. thaliana*. A phylogenetic tree for all type I and type II genes, including *C. globularis*, *S. moellendorffi*, *P. patens*, *Arabidopsis*, the fern species, and lycophytes, was constructed. MUSCLE v3.8.1551 software was used to align the sequences with default parameters (Edgar, 2004). Next, we constructed an ML phylogenetic tree via IQ-Tree v2.1.2 with 1,000 replicates (Minh et al., 2020). iTOL v6 (https://itol.embl.de) was utilized for visualizing and annotating the tree (Zhou et al., 2023). TBtools v2.034 was used to show the copy number heatmap of MADS-box subclades (Chen et al., 2020).

### 2.5 Expression patterns of MADS-box family genes in *L. japonicum*

RNA-Seq data of *L. japonicum* from nine different tissues with two biological replicates (including prothalli of 1.0, 1.5, 2.0, and 5.0 mm, trophophylls as well as rhizomes of young sporophytes, and trophophylls and sporophylls of mature sporophytes) were obtained to determine the expression patterns of MADS-box family genes in *L. japonicum* (Aya et al., 2015). TBtools v2.034 was performed to generate the heatmap of MADS-box genes (Chen et al., 2020).

### 2.6 Statistical analysis

A Kruskal-Wallis test in R v4.2.1 was used to detect the significance of differences in copy numbers of MADS-box genes among land plant groups.

## 3 Results

### 3.1 Identification and distribution of MADS-box genes in ferns

**Figure 1:**
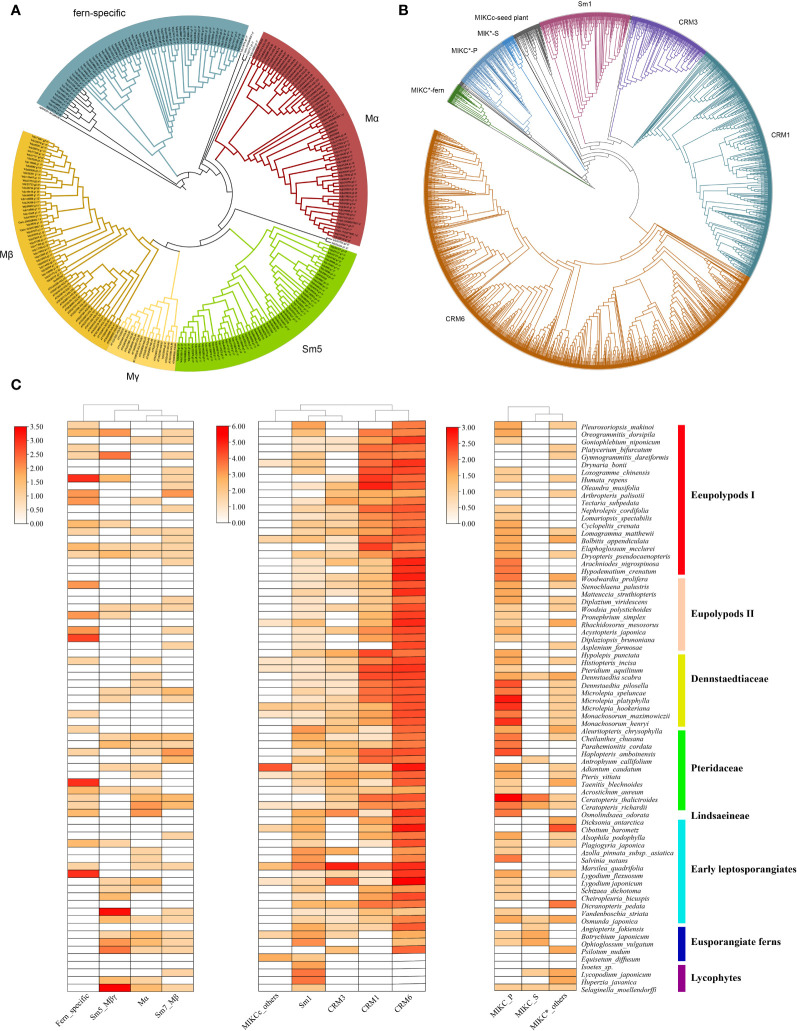
Phylogeny and distribution of MADS-box genes in extant ferns. **(A)** ML phylogeny of type I MADS-box genes from 78 plant species. CgMADS1 from *Chara globularis* was used as the outgroup. **(B)** Phylogeny of type II MADS-box genes from 78 plant species. Branches from different clades are indicated in different colors. **(C)** Distribution of MADS-box genes across 71 fern species and four lycophytes. Heatmaps for type I, MIKC^C^, and MIKC* subclades were generated using TBtools based on **Supplementary Table S1** (Chen et al., 2020). The y-axis represents the number of MADS-box genes with log2-transformed.

We conducted searches of genome and transcriptome data of 71 fern species using the Hidden Markov Model (HMM) (PF00319) and BLASTP algorithm to identify MADS-box genes. We identified a total of 2,512 MADS-box sequences after removing 50 genes that did not encode MADS-box domains (**Figure 1**, **Supplementary Table S1**). The total number of MADS-box genes in ferns was not evenly distributed, and the number of MADS-box genes ranged from 9 to 89 per species; the lowest and highest number of MADS-box genes was detected in *Equisetum diffusum* and *Marsilea quadrifolia*, respectively.

To better clarify the evolutionary relationships among MADS-box genes in ferns, phylogenetic trees were constructed using sequences of Arabidopsis and each fern species with the maximum likelihood (ML) method. We divided the identified MADS-box genes into the type I and type II clades according to the classification of these genes in *Arabidopsis*. Only 192 MADS-box genes were determined as type I genes, and the number of genes per species ranged from 0 to 10. By contrast, more than 90% (2,320) of fern MADS-box genes were classified as type II genes (including 2,133 MIKC^C^ and 187 MIKC* genes), with the gene number variation per species from 9 to 86. According to the phylogeny of type I genes (**Figure 1A**), we identified a total of four clades, including Mα, Mβ, Mβ-γ (the Sm5 clade included *SmMADS5, 7, 8, 9, 11–13*, and *17–20*), and another fern-specific clade. According to the phylogeny of type II genes (**Figure 1B**), orthologs of the floral identity genes in the A, B, C, D, and E model were not detected in the fern species, unlike MIKC^C^ genes in angiosperms; these genes in ferns could be further subdivided into four subgroups [CRM1 (532 genes), CRM3 (189 genes), CRM6 (1,181 genes), and Sm1 (189 genes)]. MIKC* genes comprised three clusters, and 123 MIKC* P-clade genes were widespread across the fern phylogeny; one separate clade comprised 48 genes, and 16 MIKC* S- clade genes were rarely observed in ferns.

### 3.2 Comparison of MADS-box genes among land plant groups

**Figure 2:**
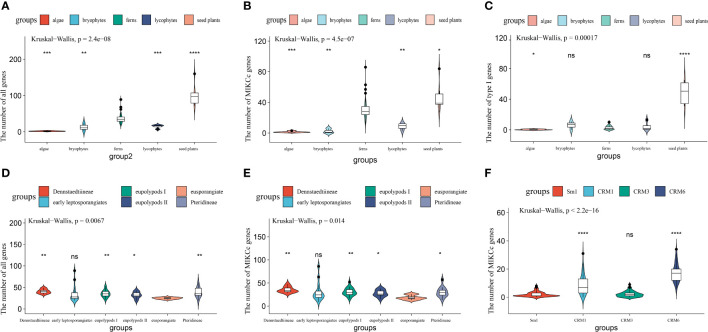
Expansion of MADS-box genes in different plant lineages. **(A–C)** Comparison of the number of all MADS-box, MIKC^C^, and type I genes among algae, bryophytes, lycophytes, ferns, and seed plants. **(D, E)** Variation in the number of MADS-box and MIKC^C^ genes among fern species. **(F)** Variation in the genes number among four subgroups of MIKC^C^ genes in Polypodiales Sample sizes (n) are as follows: algae, n=5; bryophytes, n=3; lycophytes, n=4; and ferns, n=71. Sample sizes among ferns are as follows: eusporangiate ferns, n=5; early leptosporangiates, n=14; Pteridineae, n=12; Dennstaedtiineae, n=10; eupolypods II, n=10; and eupolypods I, n=20. ns: not significant; *: p < 0.05; **: p < 0.01; ***: p < 0.001; ****: p < 0.0001..

We compared the number of MADS-box genes among land plants, including algae, bryophytes, lycophytes, ferns, and seed plants (**Figure 2**, **Supplementary Figure S1**). Only one or two MADS-box genes were present in algae, and about 20 genes were identified in *Physcomitrella patens* based on previous studies (Gramzow and Theissen, 2010; Barker and Ashton, 2013). The results show that the mean number of MADS-box genes in lycophytes was approximately 15 and ranged from 7 to 19. The number of MADS-box genes in ferns was 35 on average and ranged from 9 to 89, indicating that MADS-box genes in ferns have expanded significantly relative to lycophytes and algae (**Figure 2A**). The number of MADS-box genes was approximately 100 in seed plants and ranged from 72 in *Oryza sativa* to 160 in *Brassica rapa* (**Supplementary Table S2**).

We further detected expansions of type I, MIKC^C^, and MIKC* genes across all land plant groups. No apparent expansions of type I genes were detected in any land plant groups, with the exception of seed plants (**Figure 2C**); an average of 1–6 type I genes were identified in algae, bryophytes, lycophytes, and ferns, and 48 genes were identified in seed plants, which indicated that type I genes have undergone a significant expansion in seed plants. The average number of MIKC* genes was less than 10 for all land plant groups (**Supplementary Figure S1A**). MIKC^C^ genes have undergone a major expansion in ferns and seed plants relative to algae and lycophytes (**Figure 2B**). An average of 30 MIKC^C^ genes was present in ferns; MIKC^C^ genes were thus significantly more abundant in ferns than in algae and lycophytes. MIKC^C^ genes have undergone a significant expansion in seed plants relative to other plant groups, with an average of 47 genes.

We divided ferns into six major clades, eusporangiate ferns, early leptosporangiate ferns, Pteridineae, Dennstaedtiineae, eupolypods I, and eupolypods II as in previous reports (Shen et al., 2018), and clarified whether expansions of MADS-box genes have occurred during the evolution of ferns. We reveal that the total number of MADS-box genes was significantly higher in Pteridineae, Dennstaedtiineae, eupolypods I, and eupolypods II than in eusporangiate ferns, suggesting an expansion of MADS-box genes in Polypodiales relative to eusporangiate ferns (**Figure 2D**). MIKC^C^ genes have also undergone a large expansion in Polypodiales relative to eusporangiate ferns (**Figure 2E**), revealing that variation in the MIKC^C^ gene numbers might be the main cause of the expansion for MADS-box genes in ferns. The expansion of MIKC^C^ genes in the CRM1 and CRM6 subgroups was much larger than the expansion of the MIKC^C^ genes in the Sm1 and CRM3 subgroups in Polypodiales (**Figure 2F**).

### 3.3 Analysis of the chromosomal locations of MADS-box genes

**Figure 3:**
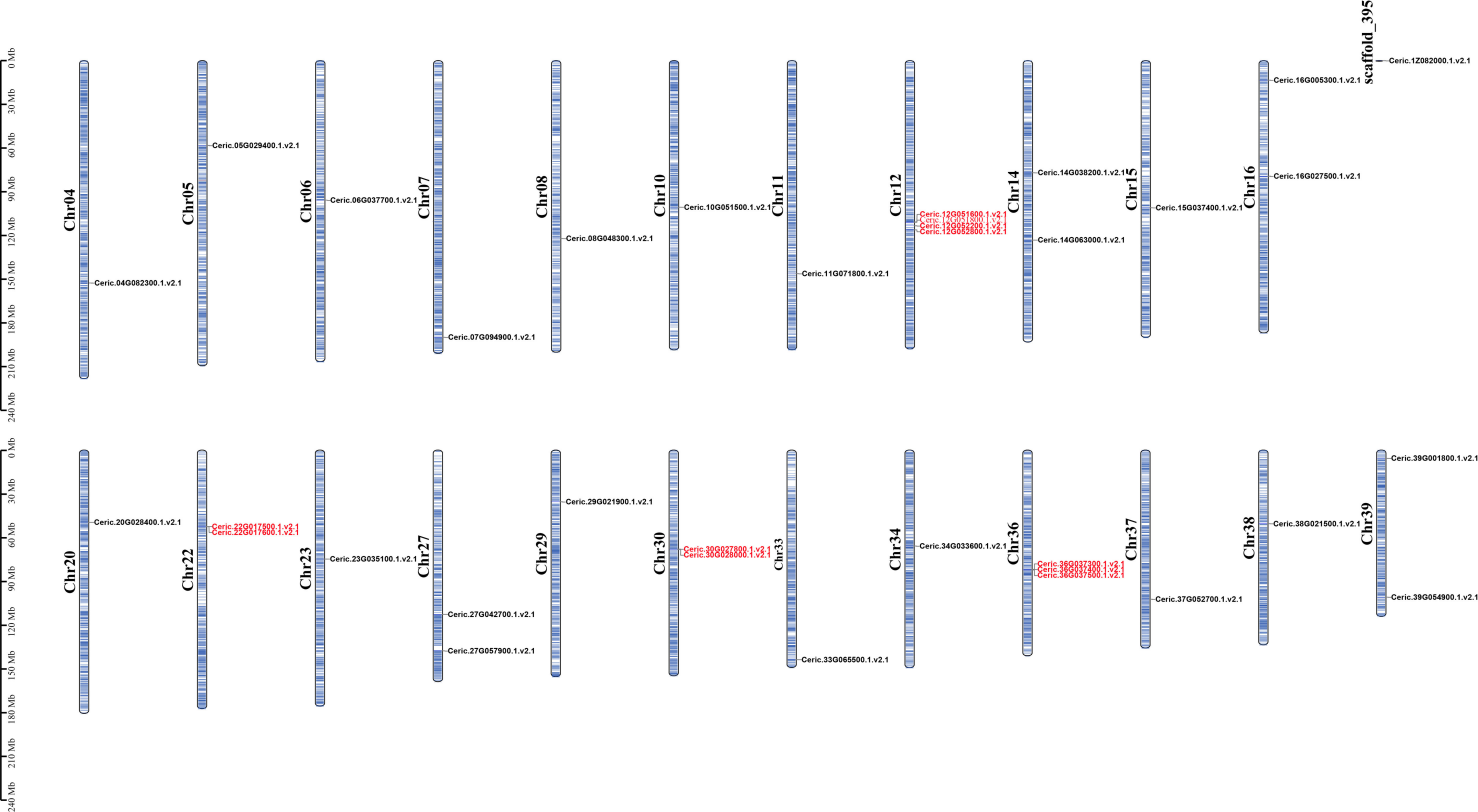
Chromosomal locations of MADS-box genes in *C. richardii*. The scale bar on the left shows the length of the chromosomes. Genes located close to Ceric.12G051600 and Ceric.12G051800, Ceric.12G051300 and Ceric.12G051700 were also considered MADS-box genes according to BLASTP searches; however, we removed these genes because they have a K domain but lack a MADS domain. The same was done for genes Ceric.36G037100 and Ceric.22G017400, which shared similar features with Ceric.12G051300 and Ceric.12G051700. Tandem duplicated genes are marked in red.

To clarify the relationship with gene expansion and tandem duplication patterns, the chromosomal locations of MADS-box genes in *C. richardii* were performed (**Figure 3**). We found that the 35 MADS-box genes were located on 23 chromosomes and ChrX of *C. richardii* unevenly. Chr12 and Chr36 contained four and three genes, respectively. Most other chromosomes contained two or fewer MADS-box genes. Chr12, Chr36, Chr22, and Chr30 contained five CRM1 and five CRM6 tandem duplicated genes, revealing that tandem duplication has contributed to the expansion of the CRM1 and CRM6 subgroups.

### 3.4 Motifs, domains and gene structure of MADS-box genes are conserved in land plants

**Figure 4:**
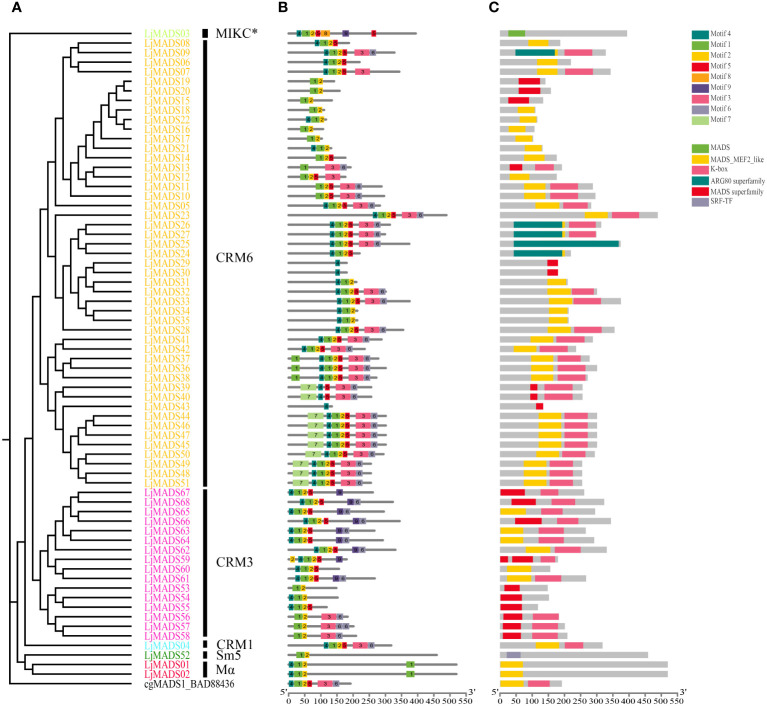
Conserved motifs and domains of 68 MADS-box proteins in *L. japonicum*. **(A)** Phylogenetic tree of *L. japonicum* MADS-box genes; **(B)** Motifs of MADS-box proteins; **(C)** Conserved domains of MADS-box proteins. The conserved motifs and domains were identified via MEME and CDD analysis, respectively. Boxes of different colors indicate different motifs or domains, and the black lines indicate non-conserved sequences. The scale indicates the protein length of the genes, and the width differences among the boxes represent the motif and domain length. There are two subgroups of MIKC-type genes, the classical MIKC-type genes (MIKCc-type genes) and a deviant type of MIKC-type genes with varible K domains (termed MIKC*-type genes).

Conserved motifs and domains were analyzed based on their phylogenetic relationships using the MEME online tool and the National Center for Biotechnology Information (NCBI) conserved domain database (CDD). A total of 10 conserved motifs were identified in land plants (**Figure 4**, **Supplementary Figure S2**). Motif 3 and 6 or motif 9 and 6 represented K domain (K1, K2, and K3 subdomains), and were shared by most MIKC^c^ proteins. Interesting, there are some genes lack a K domain, but are obviously similar to MIKC proteins, which might be related to the absence of K domain during the evolution or an artifact of the genome assembly. A total of 119 of 149 MIKC^C^ proteins had at least one motif, and the complete K domain was absent in seven *C. richardii* and 22 *L. japonicum* MIKC^C^ proteins (**Supplementary Figure S2B**). Motif 8 and 9 represented the elongated K domain of MIKC* proteins, all 32 MIKC^*^ proteins had variable K domains, with the exception of Ceric.30G028000.1.p (**Supplementary Figure S2B**). Motif 7 was specific to CRM6 subgroup proteins for *L. japonicum*, which were located at the N-terminal part before the MADS domain (**Supplementary Figure S2B**). In contrast to type II proteins, motif 10 was specific to some Mβ proteins. Besides, all MADS-box proteins contained a highly conserved MADS domain, which corresponds to motifs 1, 2, and 4 (**Supplementary Figure S2**). Among the three motifs of MADS domain, motif 1 is the central part, and it was highly conserved and present in 171 out of 181 type II and 79 out of 85 type I genes, respectively; this motif corresponds to an α-helix that contains a nuclear localization signal; Motif 4 corresponds to the N-terminal extension, and it represents the second highly conserved motif besides motif 1; Motif 2 corresponds to a β-sheet present in most Mα proteins and is relatively variable. Additionally, Motif 5 corresponds to an intervening domain (I) present in most Mα proteins of *Arabidopsis* and type II proteins.

**Figure 5:**
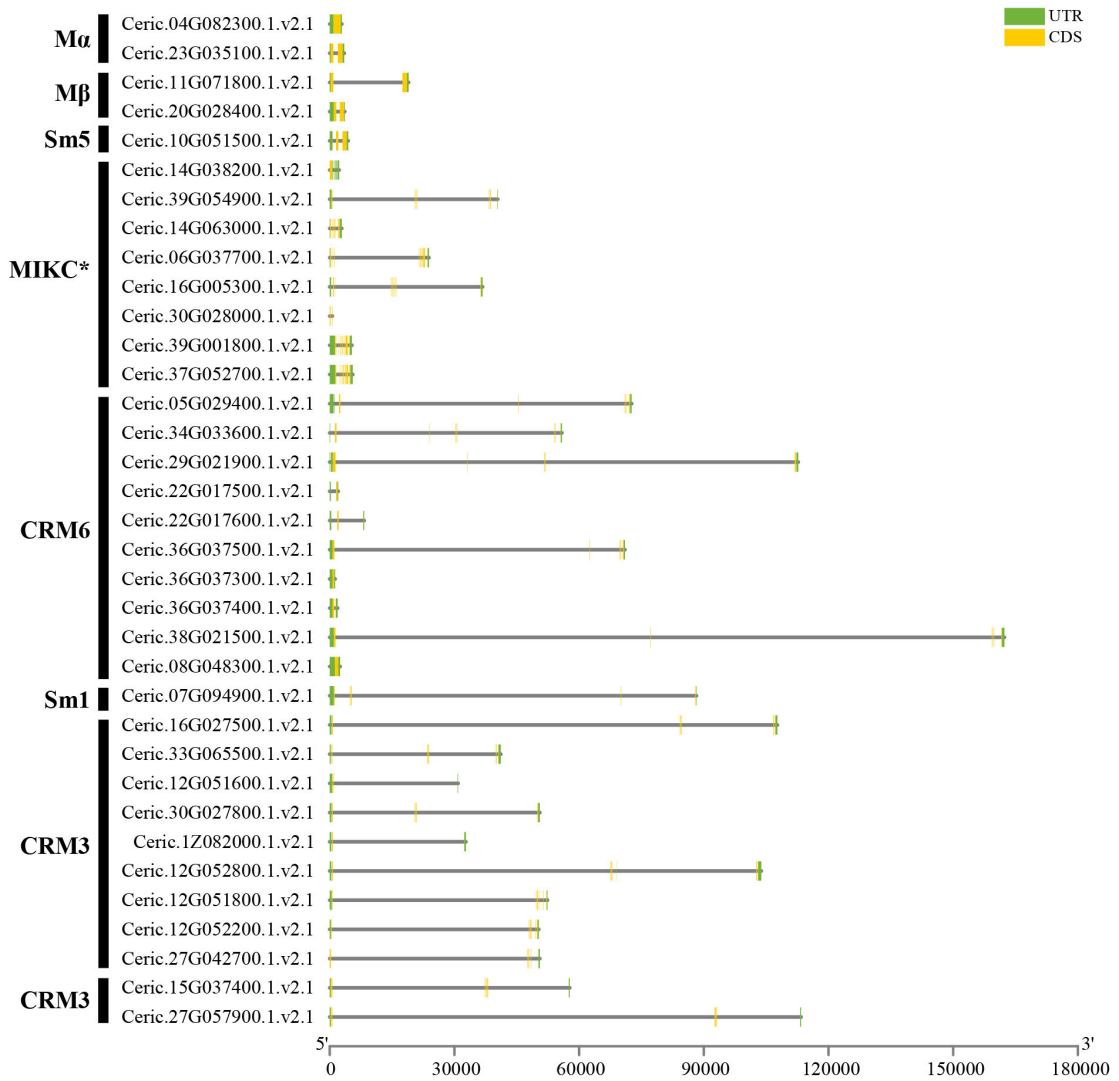
Gene structure analysis of MADS-box family genes in *C. richardii*. The UTR, exons, and introns are indicated by green boxes, yellow boxes, and solid lines, respectively. The scale indicates the gene length of MADS-box genes.

We conducted the exon–intron structure for all MADS-box genes of *Arabidopsis* and *C. richardii* to clarify differences between ferns and other plant groups. Genes exhibited similar exon–intron structures within the same group (**Figure 5**, **Supplementary Figure S3**). Type I genes for both species had one to three exons, whereas MIKC^*^ genes had seven to eleven exons; MIKC^C^ genes in *Arabidopsis* and most genes in *C. richardii* had five to nine exons. The protein length of MIKC^C^-type proteins varied from 200 to 600 amino acids; however, the gene length of MIKC^C^-type proteins were ca. 6,000 bp in AT5G10140 and 180 kb in Ceric.38G021500, which was 30-fold higher in *C. richardii* than in *Arabidopsis*.

Overall, the motifs and domains were relatively conserved, and exon–intron structures were similar in the same phylogenetic subgroup; however, incomplete motifs and gene length variation have been observed in ferns.

### 3.5 Differential expression of MADS genes in *L. japonicum*

**Figure 6:**
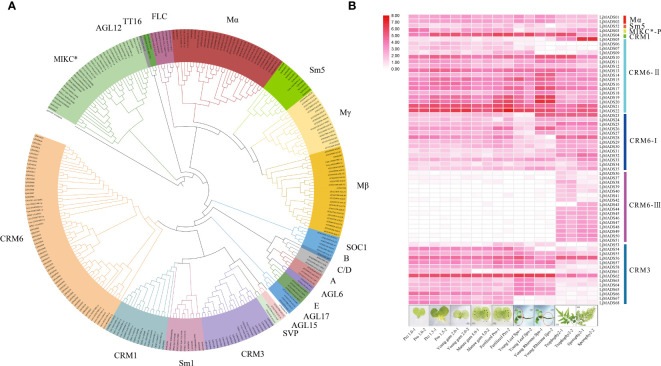
Phylogenetic tree and expression patterns of MADS-box genes. **(A)**. Phylogenetic analysis of MADS-box proteins. Different clades are indicated by different colors. The plant species included are as follows: *Arabidopsis*, *Ceratopteris richardii* (Ceric), *Lygodium japonicum* (Lj), *Selaginella moellendorffii* (Sm)*, Physcomitrella patens* (Pp), and *Chara globularis* (Cg). **(B)** Expression profiles of 68 MADS-box genes in *L. japonicum* with different subclades at the right. The RNA-Seq data were obtained from Aya et al. (2015). The heatmap was generated using log_2_(FPKM>1) expression values. Ppro, gam, and fertilized correspond to prothalli, gametophyte, and fertilized gametophyte, respectively.

To further clarify the functions of MADS-box family genes in ferns, we investigated the expression patterns of all MADS-box genes in different organs and development stages of *L. japonicum* blades using RNA-Seq data (**Supplementary Table S3**) (Aya et al., 2015). The heatmap results indicated that MADS-box genes belong to the same subgroups have similar expression patterns (**Figure 6B**). Type I genes, including *LjMADS01*, *LjMADS02*, and *LjMADS52*, were abundant in most tissues. The MIKC^*^ gene *LjMADS03* was preferentially expressed in the gametophyte, indicating that it has a conserved function in gametophyte development. The genes encoding MIKC^C^-type proteins in *L. japonicum* show highly diverse expression patterns. A total of 64 MADS-box genes were classified as MIKC^C^-type genes, and these could be further divided into CRM1-like, CRM3-like, and CRM6-like genes. *LjMADS04* is a CRM1 gene that is ubiquitously expressed in all tissues, with the exception of the sporophylls. A total of 12 of 16 CRM3 genes (*LjMADS53*–*68*) were highly expressed in the gametophyte or young sporophyte, and the expression of these genes was low or undetected in the trophophylls and sporophylls of mature sporophytes. *LjMADS56* and *LjMADS62* were widely expressed in all organs; the expression of *LjMADS59* and *LjMADS60* was too low to be detected, suggesting that they might be pseudogenes. The CRM6-like clade could be further subdivided into the CRM6-I, CRM6-II, and CRM6-III subclades (**Figure 6A**). A total of 13 genes (*LjMADS23*–*35*) were clustered with CRM6 genes (CRM6-I), and they were preferentially expressed in the trophophylls, sporophylls, and rhizomes of young sporophytes. A total of 18 of 47 genes (*LjMADS05*–*22*) were clustered with CRM7 genes (CRM6-II); *LjMADS05* was preferentially expressed in sporophyll tissue, and other genes were preferentially expressed in fertilized gametophytes or young sporophytes. The remaining 16 genes (*LjMADS36*–*51*) comprised an independent lineage (CRM6-III), and they were expressed exclusively in the trophophylls or sporophylls. The diverse gene expression patterns of MIKC^C^-type proteins providing clues that they might be functionally diverse across both haploid and diploid generations.

## 4 Discussion

### 4.1 MADS-box genes in land plants are highly conserved

MADS-domain transcription factors are widespread in eukaryotes; they all possess conserved MADS domains. These genes can be classified into type I and type II two groups according to their phylogenetic relationships, domains, and exon–intron structure (Alvarez-Buylla et al., 2000; Gramzow and Theissen, 2010; Theißen and Gramzow, 2016). We identified a total of 2,512 MADS-box sequences from 71 fern species; type I and type II genes were found in bryophytes, ferns and lycophytes, and seed plants; the presence of type I and type II genes, including MIKC^C^ and MIKC* genes, in extant bryophytes and vascular plants (**Figures 1A, B**) is in agreement with the results of previous studies (Gramzow and Theissen, 2010; Thangavel and Nayar, 2018). Analysis of domains and conserved motifs in various plant groups were performed (**Figure 4**, **Supplementary Figure S2**). Consistent with previous studies, the crystal structures of the MADS domain revealed N-terminal extensions, followed by an α-helix and two antiparallel β-strands, and these regions were referred to as motif 4, 1 and 2 in our MEME motif analysis, respectively (Kaufmann et al., 2005; Theißen and Gramzow, 2016). We found that all type I and type II MADS-box proteins contained a most highly conserved MADS domain; the α-helix or motif 1 was the central and most conserved part of the MADS domain, and might be related to its essential roles in DNA binding, protein dimerization, as well as nuclear localization (Gramzow and Theissen, 2010; Theißen and Gramzow, 2016; Qiu et al., 2023); As the second highly conserved motif in MADS domain, motif 4 is also necessary and can recognize and bind to CArG boxes of target genes, including other MADS domain proteins based on CC(A/T)_6_GG for SRF-like proteins and other similar sequences (e.g., N10-type CArG box or C(A/T)_8_G for MEFE-like proteins) (Kaufmann et al., 2005; Liu et al., 2013; Theißen and Gramzow, 2016). Type II genes encode I, K, and C domains followed by the M domain. The K domain has two amphipathic α-helices with a separation of a rigid kink, which corresponds to the K1, K2, and K3 subdomains as reported in Kaufmann et al. (2005). Motifs 3 or 9 and 6 correspond to the two amphipathic α-helices of the K domain and are moderately conserved, and 114 and five of 149 MIKC^C^ proteins had the complete or partial K domain (both two or one motif), respectively (**Supplementary Figure S2B**). A total of 31 out of 32 MIKC^*^ proteins had variable K domains (**Supplementary Figure S2B**). Our findings indicate that the K domain is conserved in type II MADS-box proteins, which might play a key role in dimerization and tetramerization activities in ferns, or even the formation of higher order (multimeric) protein complexes for the regulation of different developmental processes in complex gene regulatory networks according to previous studies (Yang and Jack, 2004; Puranik et al., 2014; Theißen and Gramzow, 2016).

The exon–intron structure analysis revealed that type I genes of both *C. richardii* and *Arabidopsis* had one to three exons, which coincides with the previous studies of plant type I genes (**Figure 5**, **Supplementary Figure S3**) (De Bodt et al., 2003; Gramzow and Theissen, 2010). MIKC^C^ and MIKC^*^-type proteins have seven to eleven and five to nine exons in *C. richardii* and *Arabidopsis*, respectively (**Figure 5**, **Supplementary Figure S3**), which is consistent with the previous reports that most type II genes have seven exons on average (De Bodt et al., 2003; Gramzow and Theissen, 2010). There are some exceptions, such as a few MIKC^C^-type proteins in *C. richardii* that only have one to two exons and lack K domains; however, whether the incomplete sequences were an artifact stemming from the incomplete genome assembly or incomplete sequences during the evolution remains unclear. The gene length of MIKC^C^-type proteins was 30-fold longer in *C. richardii* (Ceric.38G021500) than in *Arabidopsis* (AT5G10140), and this might be associated with the larger genome size of homosporous ferns compared with seed plants due to the impact of transposable elements and longer introns (Marchant et al., 2022). Admittedly, seed plants and ferns had similar exon–intron structures, indicating that they were conserved.

Analysis of tissue-specific gene expression patterns of MADS-box genes in *L. japonicum* was conducted to clarify their potential functions in ferns (**Figure 6B**). MIKC* genes are expressed in pollen or the gametophyte of *A. thaliana*, rice, and bryophytes, and they have been shown to play conserved roles in the gametophyte generation of land plants using forward and reverse genetic analyses (Verelst et al., 2007; Gramzow and Theissen, 2010; Zobell et al., 2010; Kwantes et al., 2012; Liu et al., 2013). We show that *LjMADS03*, which encodes a MIKC^*^-type protein, was preferentially expressed in the gametophyte, and its expression pattern was similar to that observed in previous studies of *C. richardii*, which provides further support for its ancestral function in the haploid phase (Kwantes et al., 2012). MIKC^C^-type genes have been extensively investigated because of their roles in flower development. The ABCDE functions in flower organ specification are highly conserved within flowering plants, although some variation has been observed (Theissen et al., 2000; Kater et al., 2006; Causier et al., 2010; Gramzow and Theissen, 2010). In this study, we show that MIKC^C^-type genes in ferns comprise four subgroups, CRM1, CRM3, CRM6, and Sm1, and no orthologs of the floral homeotic genes were identified in ferns (**Figure 1B**). We found that *LjMADS04* in the CRM1 subgroup was ubiquitously expressed in all tissues (**Figures 6A, B**); this is consistent with the expression of this gene in the fern *C. richardii* (Münster et al., 1997). Most CRM3-like genes were highly expressed in the gametophyte and young sporophytes, but their expression was too low to be detected in the trophophylls and sporophylls of mature sporophytes. Hasebe et al. (1998) show that CRM6 proteins obtained additional N-terminal amino acids and appear to be considerably close to the AG clade of seed plants. Whereas we show that they do not share similar sequence at the elongated N-terminal amino acids, and have not been shown to be orthologs, so they might be two independent clades. Amazing, 10 of 15 CRM6-III genes in *L. japonicum* with the specific motif 7 at the N-terminal of the MADS domain were preferentially or exclusively expressed in the tissues of trophophylls and sporophylls (**Figures 4**, **6B**), and the expression of CRM6-like genes VsMB6 proteins in the fern species *Vandenboschia speciosa* is significantly higher in the sporophyte than gametophyte, which may have been recruited and involved in the specification of organs similar to floral homeotic genes in seed plants (Yanofsky et al., 1990; Theissen et al., 2000; Ruiz-Estévez et al., 2017). In additional, some MIKC^C^-type genes in angiosperms play key roles in the development of reproductive structures, including flowering time determination, fruit formation, and seed development. Hence, MIKC^C^-type genes likely play key and relatively conserved functions in diverse aspects of diploid sporophyte development, especially reproductive structures, in extant ferns and seed plants (Gramzow and Theissen, 2010; Gramzow et al., 2012).

Generally speaking, the gene types encoding MIKC proteins, especially those encoded by MIKC* genes, as well as their domains, motifs, and expression patterns, are conserved. MIKC^C^ genes in extant ferns have acquired diverse expression patterns over evolutionary time, providing clues for their relatively conserved functions regulating diverse aspects of diploid sporophyte development.

### 4.2 Expansion and diversification of MADS-box genes

MADS-box proteins are important transcription factors, and prevalent in almost all eukaryotes, including plants, animals, as well as fungi (Norman et al., 1988; Passmore et al., 1988; Sommer et al., 1990; Yanofsky et al., 1990; Gramzow and Theissen, 2010; Thangavel and Nayar, 2018). Previous studies have shown that MADS-box family genes have experienced a more rapid expansion in land plants compared with other eukaryotes. There are only 1–4 and 2–8 MADS-box genes in extant fungi and animals, respectively. However, the number of MADS-box genes increased substantially during the evolution of land plants (Theißen and Gramzow, 2016). The number of MADS-box genes per species among 71 fern species ranged from 9 to 89 (**Supplementary Table S1**). The number was significantly greater in Polypodiales (Pteridineae, Dennstaedtiineae, eupolypods I, and eupolypods II) than in eusporangiate ferns and lycophytes (**Figure 2**), which might be related to the rapid radiation of leptosporangiates and their more complex body plans (Kaufmann et al., 2005; Schuettpelz and Pryer, 2009; Thangavel and Nayar, 2018; Qiu et al., 2023).

MADS-box genes in ferns include type I and type II genes (containing MIKC^C^ and MIKC* genes) according to the phylogenetic analysis. Although an average of 48 type I genes were identified in flowering plants, only less than 10 genes were identified across 71 fern species. We detected no apparent expansions of type I genes (**Figure 2C**), and ferns exhibited a low percentage of type I genes similar to that observed in three conifer genomes, as well as in a previous study of the endangered fern *V*. *speciosa*, which might be related to the lower birth rate or higher death rate in ferns, or the lack of ovules or seeds than in angiosperms (Gramzow and Theissen, 2010; Gramzow et al., 2014; Ruiz-Estévez et al., 2017). No apparent expansions were detected for MIKC* genes (**Supplementary Figure S1A**), and low copy numbers of MIKC* genes might be associated with their conserved functions in the male gametophyte development of fern species (Gramzow and Theissen, 2010; Gramzow et al., 2012). MIKC^C^ genes are well-known floral organ development genes that play diverse roles in nearly all aspects of diploid sporophyte development (Gramzow and Theissen, 2010; Gramzow et al., 2012). We show that MIKC^C^ genes are significantly more abundant in ferns than in algae and lycophytes but less abundant in seed plants, with an average of 47 genes (**Figure 2B**); this is consistent with the morphological novelties of ferns with the dominant sporophytic generation and the formation of increasingly complex body plans (Gramzow et al., 2014; Thangavel and Nayar, 2018; Leebens-Mack et al., 2019; Qiu et al., 2023). With larger genomes and higher numbers of chromosomes than seed plants, many ferns have undergone several whole-genome duplication (WGD) events (Leebens-Mack et al., 2019; Marchant et al., 2022). Four CRM1 genes were preferentially retained following WGD events in *C. thalictroides* (Zhang et al., 2019). Moreover, we observed that 29 of 35 MADS-box genes in *C. richardii* were MIKC^C^ genes, and 11 were derived from tandem duplications (**Figure 3**); thus, whole-genome duplications and tandem gene duplications have had major effects on the expansion and evolution of MIKC^C^ genes. The expanded MIKC^C^ genes might acquire novel functions via neofunctionalization or subfunctionalization and allow these genes to participate in intricate gene regulatory networks and promoted survival in variable environments (Theißen and Gramzow, 2016; Thangavel and Nayar, 2018; Zhang et al., 2019).

### 4.3 MADS-box genes in the MRCA of extant ferns

Mα and Mβ/γ genes were probably appeared in the most recent common ancestor (MRCA) of extant bryophytes and vascular plants (Gramzow et al., 2012). Our findings indicate that ferns have Mα (*SmMADS14*, *15*), Mβ (Sm*7* clade including *SmMADS7*), and Mβ-γ (Sm5 clade including *SmMADS5, 7, 8, 9, 11*–*13*, *17*–*20*) genes, as well as 65 fern-specific genes clustered into an individual clade (**Figure 1A**), suggesting that the MRCA of extant lycophytes and ferns had at least three type I genes.

The MRCA of extant spermatophytes had probably 11 seed plant-specific MIKC^C^ superclades, and a total of 17 or at least 12–14 MIKC^C^ genes were present in the MRCA of gymnosperms or angiosperms, respectively (Gramzow et al., 2014; Theißen and Gramzow, 2016). Our phylogenetic analysis revealed at least four subfamilies of MIKC^C^ proteins (CRM1, CRM3, CRM6, and Sm1) in ferns (**Figures 1B**, **6A**). CRM1 subfamily genes were present in nearly all leptosporangiate ferns; they were absent in all eusporangiate ferns, with the exception of *Angiopteris fokiensis*, suggesting that CRM1 members were present in the MRCA of leptosporangiate ferns. Previous researches have indicated that CRM6-like genes are present in a few leptosporangiate ferns and only one eusporangiate fern, *Ophioglossum pendunculosum* (Münster et al., 1997; Ruiz-Estévez et al., 2017; Thangavel and Nayar, 2018); we strongly suggests that CRM6-like genes have been present in all ferns but not *E*. *diffusum*, including four eusporangiate ferns and 66 leptosporangiate ferns (**Figure 1C**, **Supplementary Table S1**). Within CRM6-like genes, CRM6 (CRM6-I) and CRM7 (CRM6-II) comprise two large sister clades that might represent two large subfamilies, and they exhibit different expression patterns (**Figures 6A, B**). The former was highly or exclusively expressed in mature sporophytes, whereas the latter was preferentially expressed in the gametophyte and young sporophytes, which might reflect their different functions and independent evolutionary histories (including duplication events); however, this clade is not strongly supported by analyses with large datasets of widely divergent taxa. CRM3 and Sm1-like genes were present in 51 and 65 of 71 ferns, respectively (**Figure 1C**, **Supplementary Table S1**). This indicates that CRM3, CRM6, and Sm1-like genes had existed in the MRCA of ferns, and might have been lost in several ferns during their evolutionary radiation; alternatively, some MADS-box genes were expressed below the detection limit because of limited sampling at specific stages.

**Figure 7:**
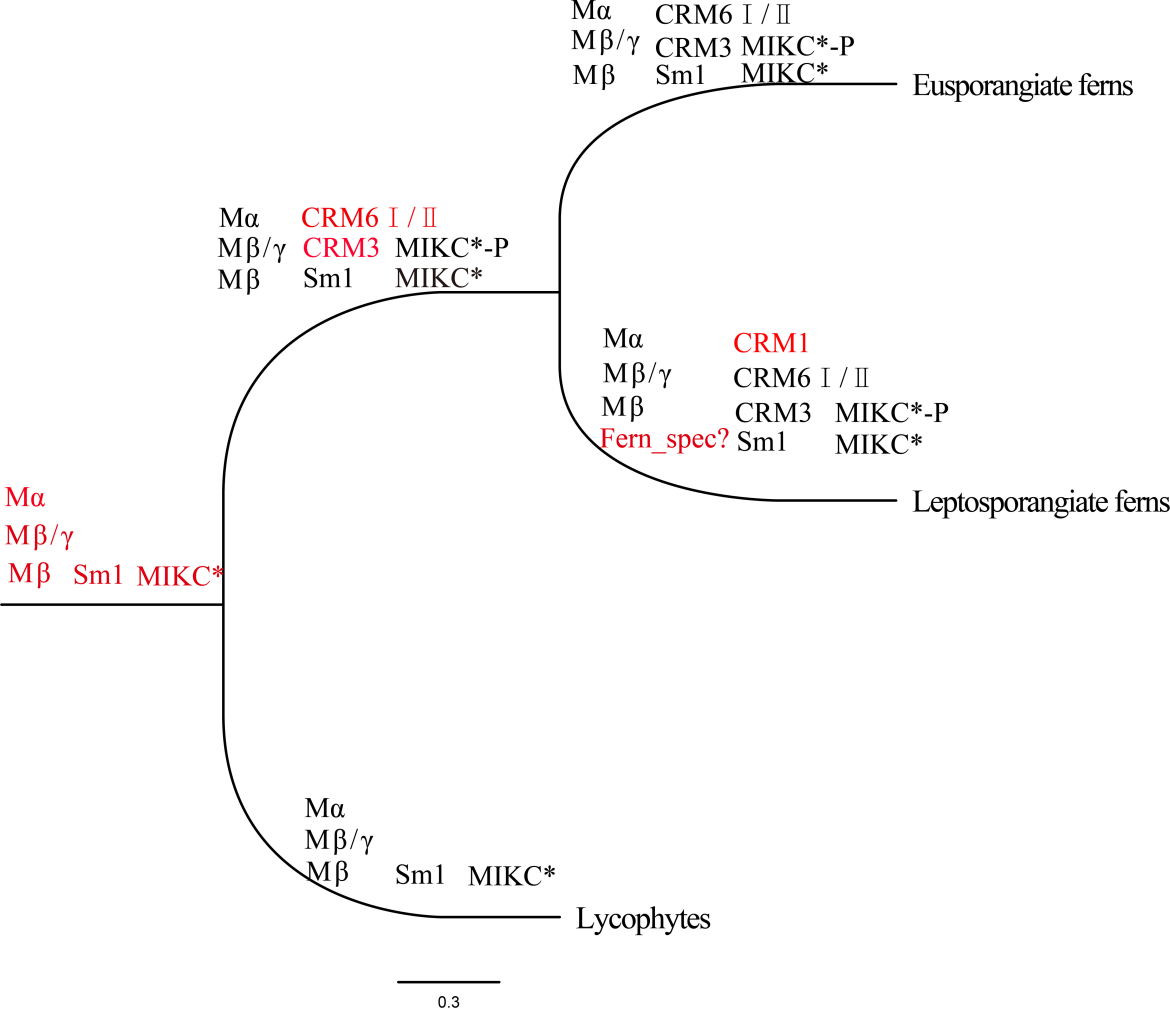
Major clades of MADS-box genes in the phylogeny of extant fern species. There are three columns at each node. The first column shows type I genes, the second column shows MIKC^C^ genes, and the third column shows MIKC* genes. Red denotes genes that were included for the first time in the current phylogeny. Clade names are based on those in previous studies, with the exception of Sm1 and Sm7. CRM6I/II indicates CRM6 and CRM7.

At least one MIKC* gene was present in the MRCA of extant bryophytes and vascular plants, and the S-clade and P-clade of MIKC* genes were found in the MRCA of ferns and vascular plants (euphyllophytes), neither in bryophytes nor lycophytes (Gramzow et al., 2012; Theißen and Gramzow, 2016). As observed in **Figure 1**, SmMADS4, as well as SMMADS10 and MIKC* of *P. patens* formed together with P-clade and S-clade with low bootstrap supports (50%), respectively. Whereas the shared clades changed in **Figure 6A** with fewer samples, the MIKC* of *P. patens* and *S. moellendorffii* clustered into bryophytes and lycophytes-MIKC* two individual clades, which is in agreement with previous studies (Kwantes et al., 2012; Liu et al., 2013). It is possible that P-clade and S-clade are closely related to the MIKC* of bryophytes and lycophytes, or large datasets with divergent taxa affect the topology. More sampling and further studies are necessary to clarify the relationship of MIKC* genes for bryophytes and lycophytes. Four *Ceratopteris* MIKC^∗^ genes, namely *CRM13*–*16*, corresponding to the S- and P-clades of MIKC^*^ proteins were identified by Kwantes et al. (2012), which suggests that the two types of MIKC^*^genes were present in the ancestor of ferns as well as seed plants. However, our phylogenetic analysis reveals that the P-clade and fern-specific MIKC^*^ proteins were present in several fern species, but S-clade MIKC^*^ proteins were only detected in eight fern species. These genes were mainly derived from the transcriptome dataset. A previous study indicates that S-clade genes, such as CRM14 and CRM15, are weakly expressed, whereas P-clade genes are considerably more abundant in both fertile and unfertile blades (Kwantes et al., 2012); however, whether S-clade MIKC^*^ proteins represent an ancient clade of MIKC^*^ proteins remains unclear, and additional work will be needed to verify this possibility. Anyhow, we found that the MRCA of ferns might have contained at least two MIKC^*^ proteins (one P-clade MIKC* protein and one fern-specific MIKC^*^ protein), and P-clade members formed homodimers and heterodimers to play conserved functions in both sporophytic and gametophytic development (Kwantes et al., 2012). So far, our findings indicate that at least 9–10 MADS-box genes were appeared in the MRCA of extant leptosporangiate ferns (**Figure 7**).

## 5 Conclusion

We identified MADS-box proteins from 71 fern species using HMMER and BLASTP. We found that at least 8–9 MADS-box genes were present in the MRCA of ferns, 3 genes already existed in the MRCA of ferns and seed plants, and no orthologs of floral identity genes were identified among the MIKC^C^ genes. Both type I and type II genes were relatively conserved among land plants. We further indicated that MIKC^C^ genes expanded significantly in ferns. Tandem duplication and diverse expression patterns of MIKC^C^ genes (CRM1 and CRM6) contributed to the expansion of MIKC^C^ genes and their functional diversification. Our findings shed new light on the evolution of MADS-box genes in ferns and their potential functions. More genome data and functional analyses are needed to further clarify the roles of MADS-box genes.

## Data availability statement

The MADS-box proteins presented in the study could be retrieved from https://doi.org/10.6084/m9.figshare.25866847.v1.

## Author contributions

RZ: Methodology, Software, Writing – original draft, Writing – review & editing, Formal analysis, Funding acquisition, Visualization. JZ: Software, Visualization, Writing – review & editing. Y-XX: Methodology, Writing – review & editing. J-MS: Writing – review & editing. S-JD: Writing – review & editing. HS: Writing – review & editing. Y-HY: Formal analysis, Funding acquisition, Writing – review & editing.
