# Supplementary material for: Dynamic evolution of MADS-box genes in extant ferns via large-scale phylogenomic analysis
Source: Front Plant Sci. 2024 Jun 21;15:1410554. doi: 10.3389/fpls.2024.1410554 (PMC11224435; doi:10.3389/fpls.2024.1410554)
Supplement: Supplementary file 1 [file DataSheet_1.zip › Figure S1. Expansion of MADS-box family genes in ferns relative to other land plants.pdf]

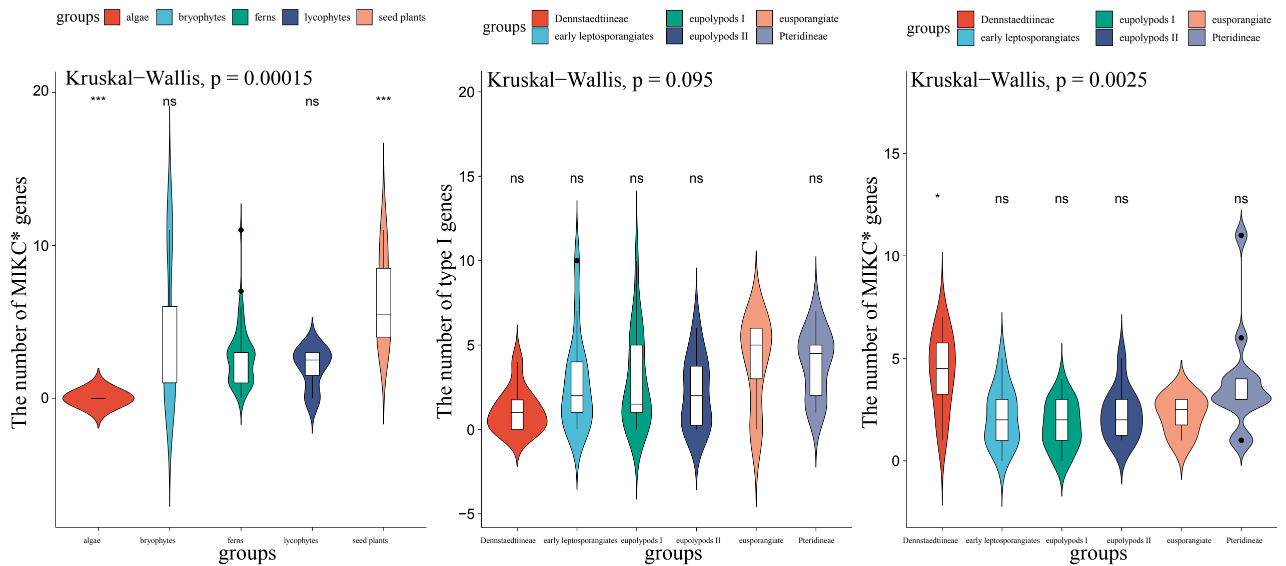

groups
algae
bryophytes
ferns
lycophytes
seed plants
Kruskal−Wallis, p = 0.00015
***
ns
ns
***
The number of MIKC* genes
20
10
0
groups
Dennstaedtiineae
early leptosporangiates
eupolypods I
eupolypods II
eusporangiate
Pteridineae
Kruskal−Wallis, p = 0.095
ns
ns
ns
ns
ns
The number of type I genes
20
15
10
5
0
−5
groups
Dennstaedtiineae
early leptosporangiates
eupolypods I
eupolypods II
eusporangiate
Pteridineae
Kruskal−Wallis, p = 0.0025
*
ns
ns
ns
ns
The number of MIKC* genes
15
10
5
0
groups
