# Supplementary figures and images for: Dynamic evolution of MADS-box genes in extant ferns via large-scale phylogenomic analysis
Source: Front Plant Sci. 2024 Jun 21;15:1410554. doi: 10.3389/fpls.2024.1410554 (PMC11224435; doi:10.3389/fpls.2024.1410554)

Type I

MIKCc

MIKC\*

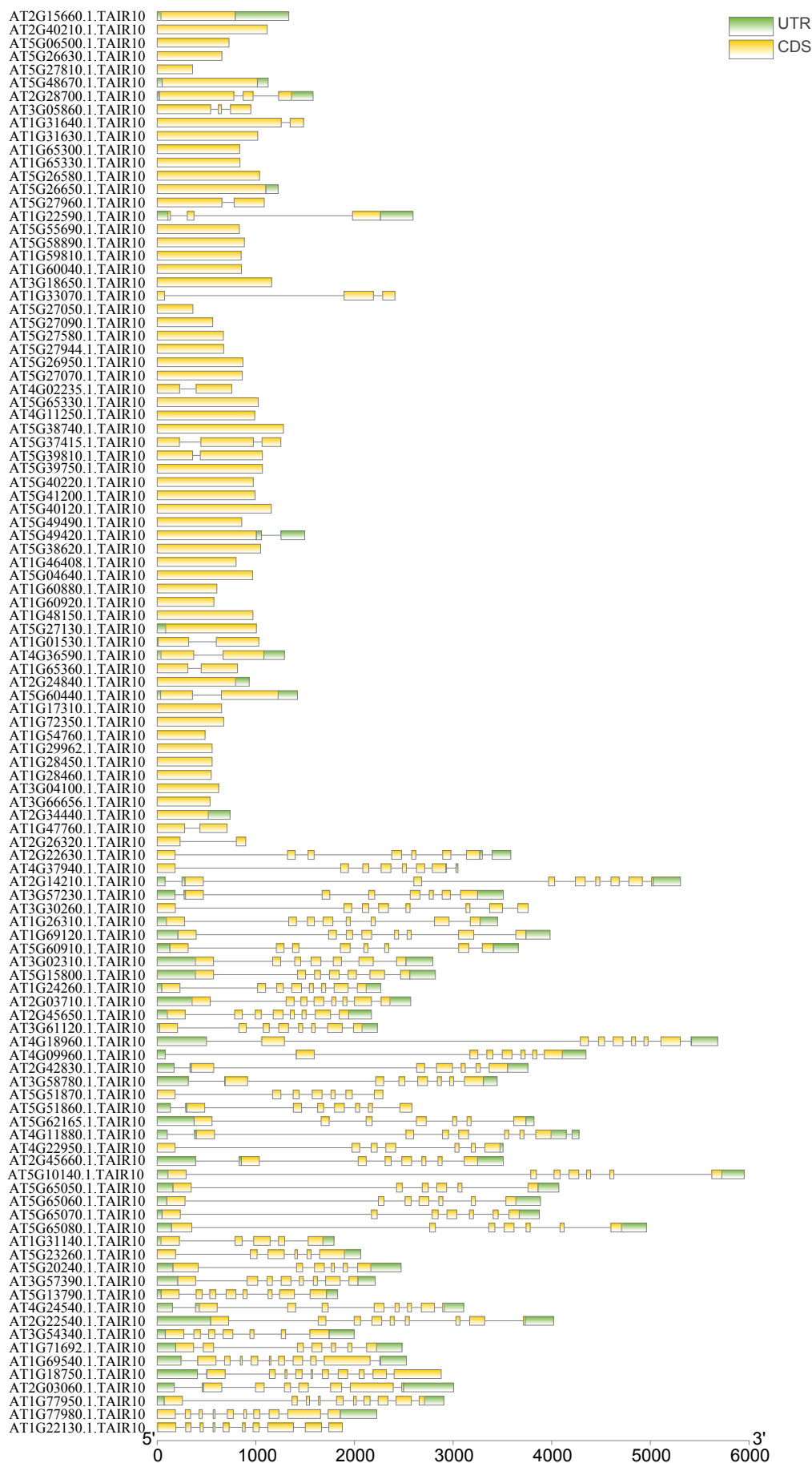

Supplement: Supplementary file 1 [file DataSheet_1.zip › Figure S3 Gene structure analysis of MADS-box family genes in Arabidopsis.pdf]

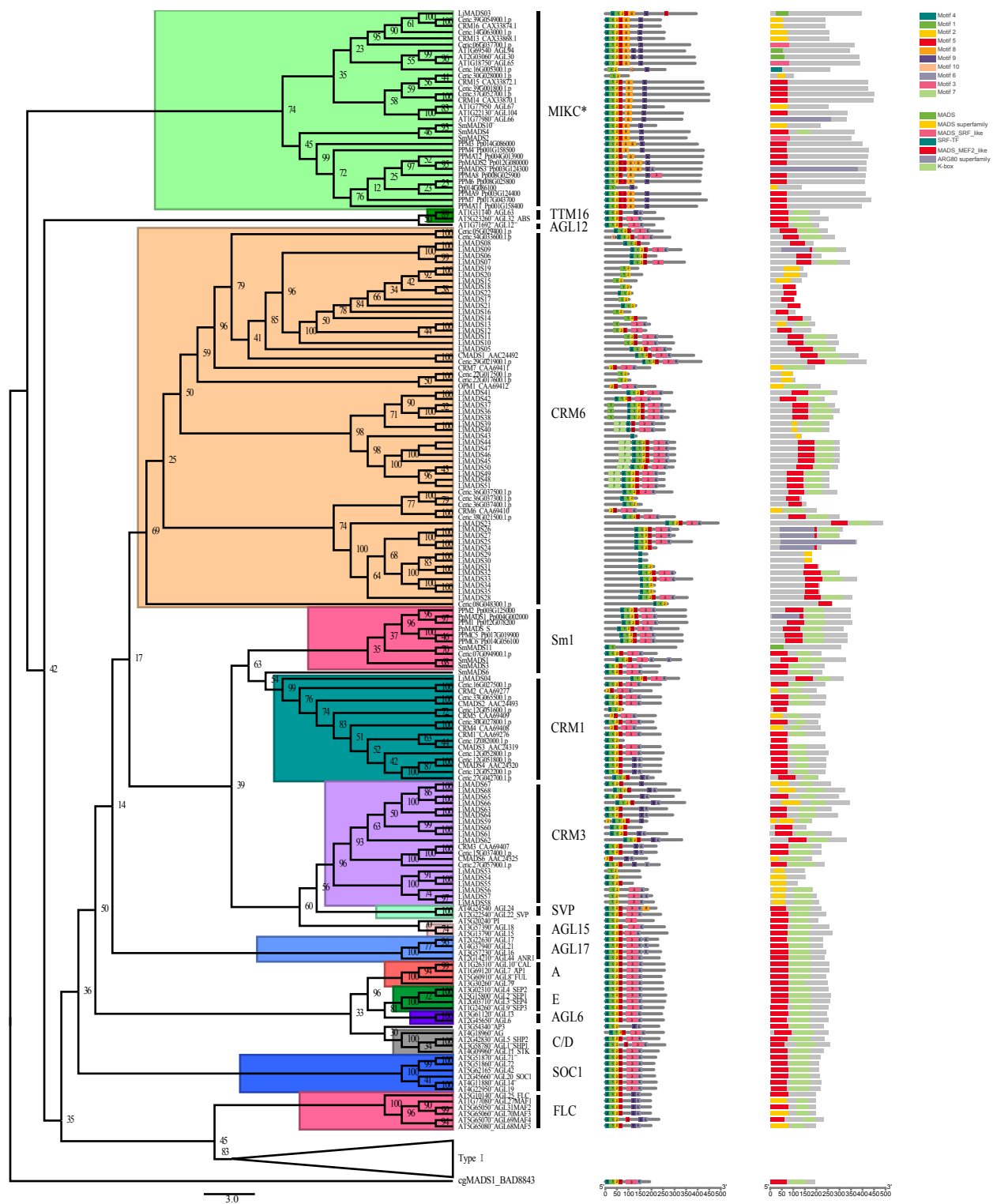

Supplement: Supplementary file 1 [file DataSheet_1.zip › Figure S2b. Conserved motifs and domains of type II MADS-box proteins for land plants.pdf]

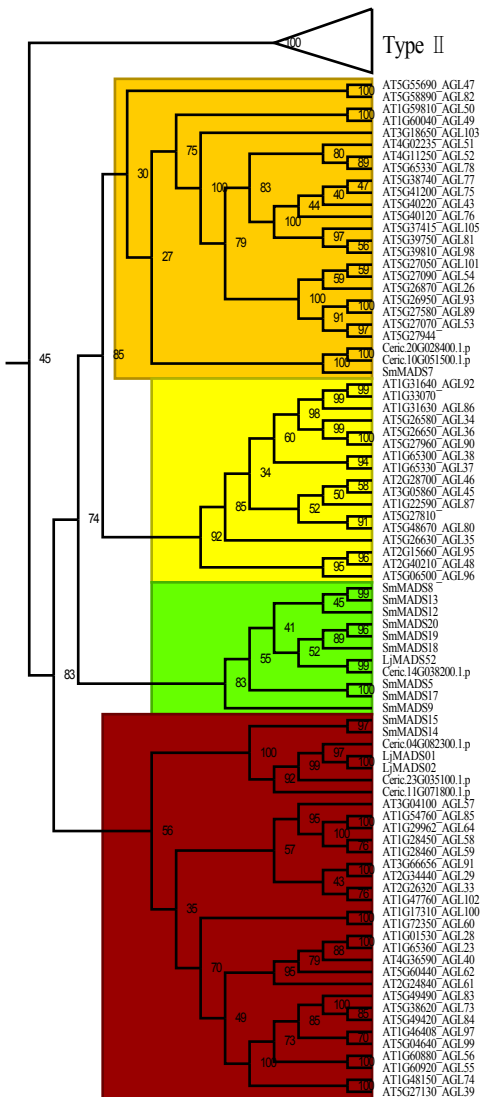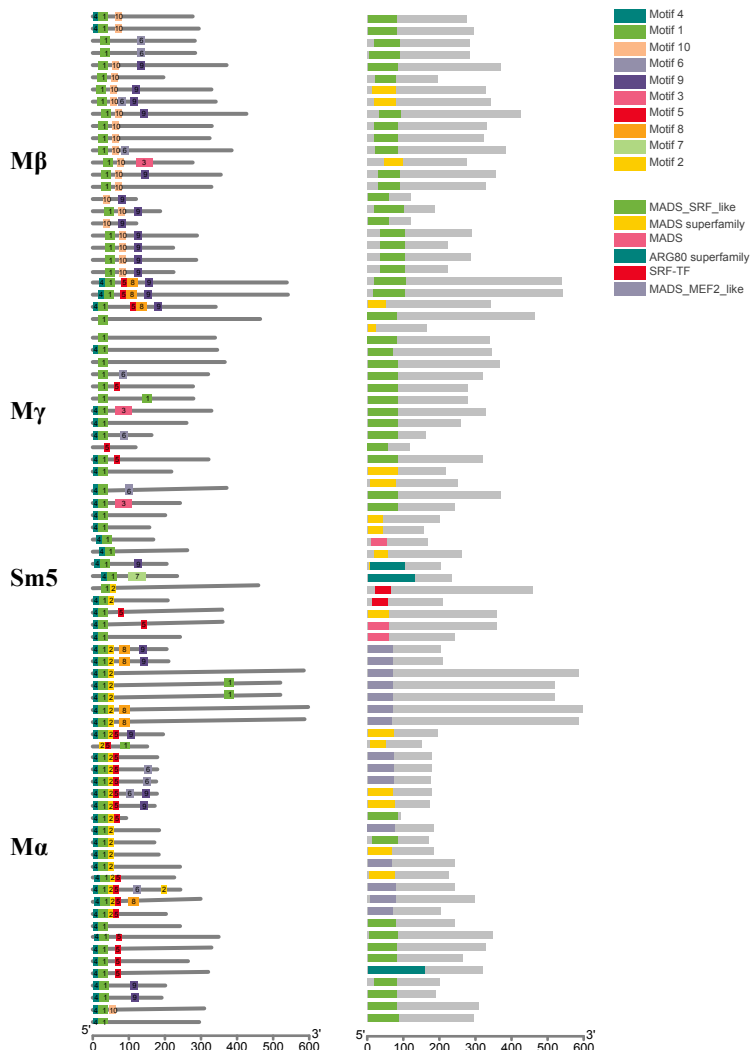

Supplement: Supplementary file 1 [file DataSheet_1.zip › Figure S2a. Conserved motifs and domains of type I MADS-box proteins in land plants.pdf]
